# Quantum imaging with a photon counting camera

**DOI:** 10.1038/s41598-022-10037-x

**Published:** 2022-05-18

**Authors:** Osian Wolley, Thomas Gregory, Sebastian Beer, Takafumi Higuchi, Miles Padgett

**Affiliations:** 1 School of Physics and Astronomy, University of Glasgow, Glasgow, UK; 2 Hamamatsu Photonics Deutschland GmbH, 82211 Herrsching am Ammersee, Germany; 3 Hamamatsu Photonics K.K, 812 Joko-cho, Higashi-ku, Hamamatsu, Shizuoka 431-3196 Japan

**Keywords:** Quantum optics, Optical physics

## Abstract

Classical light sources emit a randomly-timed stream of individual photons, the spatial distribution of which can be detected with a camera to form an image. Quantum light sources, based on parametric down conversion, emit photons as correlated photon-pairs. The spatial correlations between the photons enables imaging systems where the preferential selection of photon-pairs allows for enhancements in the noise performance over what is possible using classical light sources. However, until now the technical challenge of measuring, and correlating both photons has led to system complexity. Here we show that a camera capable of resolving the number of individual photons in each pixel of the detector array can be used to record an image formed from these photon-pair events and hence achieve a greater contrast than possible using a classical light source. We achieve an enhancement in the ratio of two-photon events compared to one-photon events using spatially correlated SPDC light compared to uncorrelated illumination by a LED. These results indicate the potential advantages of using photon counting cameras in quantum imaging schemes and these advantages will further increase as the technology is developed. Operating in photon sparse regimes such systems have potential applications in low-light microscopy and covert imaging.

## Introduction

Quantum imaging utilises correlations between entangled photon-pairs to achieve enhancements beyond classically achievable limits^1–5^. The ability to observe these correlations at the single photon level is afforded by low noise detector technologies that are sensitive to single photons. These quantum enhancements can be extracted from correlations between pairs of single photon detection events, however, photon-pairs that arrive within the same pixel and/or time bin may not be easily distinguished from a single photon event due to limitations of detector technology. The inability to make this distinction prevents the full extent of the correlations between photon-pairs from being utilised in some imaging contexts. The capacity to distinguish between a two-photon event and a one-photon event would enable an increased amount of information to be extracted from experimental data thereby improving the efficiency of quantum imaging systems. Such improvements will allow quantum imaging techniques such as quantum enhanced microscopy to be implemented in real-world applications.

A common source of entangled photon-pairs that are used in many quantum enhanced imaging experiments is spontaneous parametric down-conversion (SPDC) within a non-linear crystal. In SPDC, a photon-pair is created by the spontaneous parametric downconversion of a single pump photon and due to conservation laws the downconverted photon-pairs are strongly correlated in their position and anti-correlated in their transverse momentum. With a spatially resolving detector array it is possible to image and measure the correlations between photon-pairs realising a large number of entangled states^6–8^. Such measurements can be made due to the capability of single photon sensitive detectors to distinguish a single photon from the absence of a photon by the setting of an appropriate threshold and/or by time-gating the detector. However, due to the stochastic nature of the gain process within Electron Multiplying CCD detectors (EMCCD) and Intensified CCD detectors (ICCD) it is not possible to accurately resolve the actual numbers of photons in each pixel in a frame and as a result it is not possible to simply identify the two-photon events^9,10^. This is a disadvantage because in the case of the detector being positioned in the image plane of the downconversion source the most strongly correlated photon-pairs will arrive within the same pixel and time bin.

To date the inability to distinguish two-photon events from one-photon events has not prevented the development of quantum enhanced imaging systems using entangled photon-pair sources and single-photon-sensitive array detector technologies. Enhancements have been realised in terms of image resolution^11,12^, ghost imaging^13–16^, imaging through scattering media^17^, interaction-free ghost imaging^18^, and sub-shot-noise imaging^19–21^. Further to these applications, quantum illumination schemes that are resilient to the effects of noise and losses have been demonstrated^22–27^.

In this work we utilise a photon number resolving array detector that is based on CMOS technology which allows us to report a simpler method for the detection of correlated photon-pairs. The capability to distinguish two-photon events from one-photon events presents an advantage over the preceding technologies in imaging using SPDC photon-pairs because the photon-pairs can be detected in the same pixel in the same frame. Selecting these events allows the construction of an image consisting of two-photon events. We demonstrate an advantage in the ratio of two-photon events to one-photon events for images obtained under SPDC illumination when compared to images obtained under illumination from a LED source. This advantage is shown to be present across a range of illumination levels. In the context of quantum imaging the ability to identify two-photon events will allow the full extent of the correlations between photon-pairs to be utilised by image-plane applications in which the photon-pair events occur in the same pixel at the detector. Whilst we acknowledge the limited performance of our experiment, the method that we present method will improve efficiency with which data is collected and increase the rate at which images are populated with events, thereby increasing the opportunity for quantum imaging technologies to transition into real-world applications.

## Results

### Imaging system

**Figure 1:**
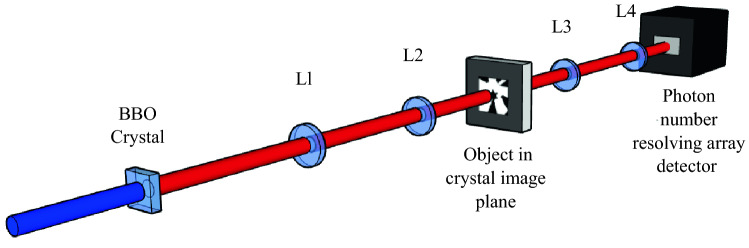
Schematic of the experimental setup. A $355 \,\text {nm}$ laser pumps a BBO crystal cut for type-I degenerate downconversion to generate downconverted photon-pairs at $710 \,\text {nm}$. Lenses $L_{1}=400 \,\text {mm and } L_{2}=100 \,\text {mm}$ demagnify the beam onto the image plane by a factor of 4 and this plane is then further demagnified onto the detector plane by a factor of 2 by lenses $L_{3}=100 \,\text {mm and } L_{4}=50 \,\text {mm}$ to give a total demagnification of the beam by a factor of 8 and image the spoke object located in the image plane of the crystal onto the camera.

The experimental setup used is shown in Fig. 1. A laser with output at $355 \,\text {nm }$ is collimated and expanded to a $\sim 10\, \text {mm}$ diameter beam before pumping a $1 \,\text { mm}$ thick BBO crystal cut for type-I parametric down conversion. This generates SPDC photon-pairs of degenerate wavelength centred at $710 \,\text {nm}$. A pair of dichroic interference filters placed after the downconversion source are used to remove the pump and a $10 \,\text {nm}$ bandpass interference filter centred at $710 \,\text { nm}$ is positioned on the camera to select only the SPDC photon-pairs. The camera used is a scientific CMOS camera, the Hamamatsu ORCA-Quest. The design of the readout circuitry combined with the low readout noise of the ORCA-Quest allow for the resolution of the actual number of photons detected in a pixel. The SPDC beam is demagnified by a factor of 4 using lenses $L_{1}=400\, \text {mm and } L_{2}=100 \,\text {mm}$ onto an image plane in which a binary spoke target is located. This plane is demagnified by a factor of 2 onto the array detector by lenses $L_{3}=100\,\text {mm and } L_{4}=50\,\text {mm}$ to give a total demagnification of the SPDC beam by a factor of 8. A LED emitting light at $660 \text { nm}$ in the same imaging system is used as the classical reference source to which we compare the performance of the down-conversion source.

We choose the de-magnification and crystal length of our system such that the number of pairs detected in the same pixel is maximised. The correlation strength $\sigma _c$ in the image plane of the crystal is given by

1 $$\begin{aligned} \sigma _c = \sqrt{\alpha L \lambda_p/ 2 \pi }, \end{aligned}$$

where *L* is the length of the crystal, $\lambda_p$ the wavelength of the pump, and α = 0.455  is a constant included to account for certain approximations made in the calculation^6,7,28^. For the system presented here, with a camera with a pixel size of $4.6\,\upmu \text {m}$ this gives a correlation strength of 1.10 pixels. We choose to de-magnify the beam by a factor of 8 with our lens configuration, such that the extent of the correlations imag ed onto the camera is 0.14 pixels outwith PSF broadening effects of the imaging system. The qCMOS image sensor employs a trench structure on a $1 \times 1$ pixel basis to suppress crosstalk between pixels and as a consequence these effects are not considered in our analysis.

### Photon thresholding

**Figure 2:**
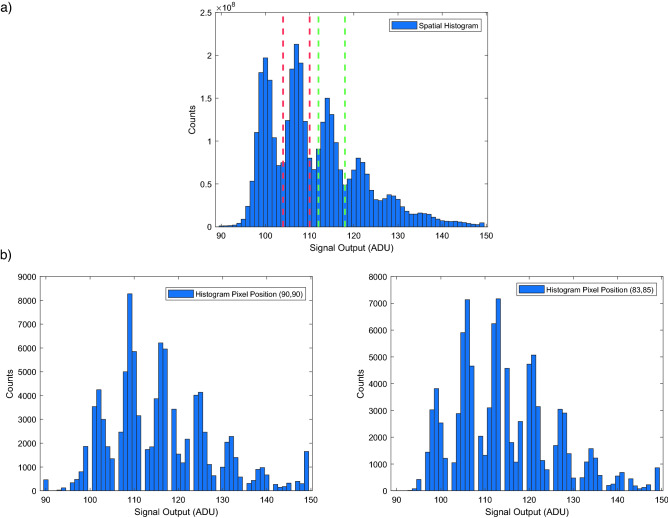
Histogram for photon number thresholds. (**a**) Spatial histogram taken for the region of interest used with the sensor illuminated by a LED source over 100,000 frames. The dashed lines indicate regions where the events with ADU signal outputs between the dashed red lines may be labelled as one-photon events and ADU signal outputs between the green dashed lines may be labelled as two-photon events. The peak preceding to the red dashed line represents pixels for which zero-photon events are detected and is present due to an offset and a non-zero readout noise. Further peaks beyond the green dashed line are present representing > two-photon events. (**b**) A selection of histograms for individual pixels from the above dataset. Histograms generated using frames obtained under a level of illumination greater than that under which the imaging data was obtained.

The ability to distinguish the number of photon events occurring in a pixel is crucial to our experiment. The signal output of the camera is digitised into ADU (analogue–digital units), from which a histogram of events can be built up in order to set thresholds corresponding to the number of photon events. For a histogram constructed over events on all pixels in the region of interest a series of global thresholds can be determined. An example of a global histogram constructed using all pixels in the region of interest over 100,000 frames can be seen in Fig. 2a. However, unlike in the case of a CCD detector in which all pixels are read out through the same readout electronics, pixels in a CMOS detector are read out individually and therefore each pixel will exhibit slightly different properties in terms of offset, gain, and noise. Differences in the readout may be seen for the individual histograms of a number of example pixels from the region of interest over 100,000 frames as can be seen in Fig. 2b ($2 \times$ temporal histograms). Within these temporal histograms there exist ADU values with zero counts, this results from a digitisation step during the readout process which leads to some values being unobtainable. These values are different for each pixel and therefore do not present in the spatial histogram.

The offset of each pixel is determined as its average value under dark conditions. The gain of the sensor is determined by measuring the mean and variance in pixel output for series of images acquired under different intensities of poisson distributed light. For the camera used in this experiment the relation between ADU and electrons for the complete sensor was determined to be 1 ADU equals $\sim 0.12$ photoelectrons. The precise gain of each pixel is derived from the shape of its temporal histogram under illumination by counting the ADUs per electron peak. Measuring the precise gain on a per pixel basis enables corrections to achieve a uniform sensor response. With gain and offset, the value of each pixel can be expressed in photoelectrons. In a straightforward approach, each pixel with a signal $e^{-}< 0.5$ electrons could be assigned to 0 photons, each pixel with $0.5<e^{-}< 1.5$ electrons to 1 photon, each pixel with $1.5<e^{-}< 2.5$ electrons to 2 photons, and so on. While this approach produces acceptable results, the pixel specific readout noise is ignored, and noisier than average pixels will result in a large number of false positives being registered. In order to prevent this, a thresholding method based on maximum likelihood is chosen.

The probability of the output of a specific pixel depends on the input photon statistics as well as on the readout noise of that specific pixel. While the readout noise is typically a continuous gaussian distribution (which is digitised in a later step), the photon distribution is discrete in nature and depends on the source. The total output probability function will be a convolution of the photon statistics with the readout noise of the specific pixel. This method needs as input parameters the readout noise of the pixels and the photon distribution. The readout noise is derived from dark frames, while the photon distribution is estimated empirically from 10 pixels with a below average noise value (around $0.18 e^{-}$). Some pixels exhibit a noise profile which deviates from a gaussian shape, those pixels amount to 122 of the 5103 pixels, or $2.4\%$, of all pixels in the region of interest and were excluded from further analysis.

**Figure 3:**
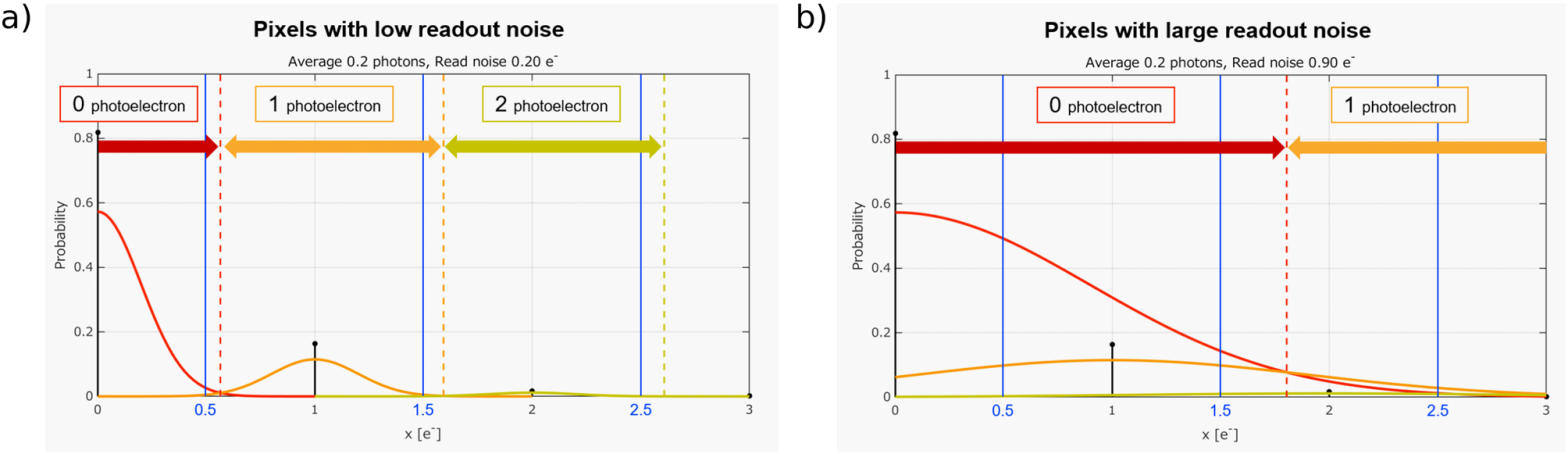
Maximum likelihood photon number thresholding method. An example of the maximum likelihood method to estimate the number of photons in a pixel for (**a**) pixels with low readout noise, and (**b**) pixels with a large readout noise. In the case of the pixel with a low readout noise the peaks corresponding to different photon numbers are well resolved and thresholds to distinguish the number of photon events that were detected by that pixel can be well estimated using the maximum likelihood method. In the case of pixels with high readout noise the thresholds are set higher than for those pixels with a low readout noise through the implementation of the maximum likelihood method. Implementing this method that considers the pixel readout noise reduces the number of false positives for one-photon and two-photon events, however, this will also result in an increase in the number of false negatives for these events as can be seen in the overlap of the red (zero-photon events) and orange (one-photon event) curves in (**b**).

Such a scenario is indicated in Fig. 3 for an example of a poissonian photon source with an average signal of $0.2 e^{-}$ per measurement and 2 different readout noise values, $0.2 e^{-}$ and $0.9 e^{-}$. The solid blue lines indicate the aforementioned half marks between the photoelectron events, while the dashed lines indicate the thresholds based on maximum likelihood. In the case of low signal intensities ($< 1e^{-}$ per pixel per measurement), pixels with a higher readout noise will be assigned higher thresholds using this method, thereby more effectively avoiding false positives for both one photon events and two photon events. However, it is also the case that there will be an increase in the number of false negatives for the one-photon and two-photon events but a false negative does not degrade the resulting image as is the case for a false positive event.

### Photon number resolved imaging

**Figure 4:**
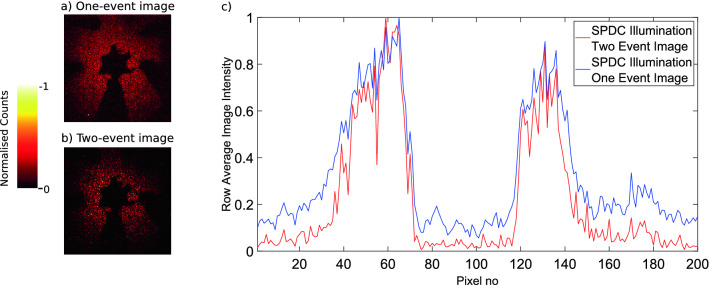
One-photon event image against a two-photon event image. Images consisting of (**a**) one-photon events and (**b**) two-photon events of a binary star target acquired over 10,000 frames. The object is illuminated by the SPDC source at a light level where the number of photon illumination events is 4.70 times the dark noise events of the camera. Each image is normalised independently to allow representation on the same scale. Also shown is a cross-section of image intensity against pixel number (**c**), for the one-photon (blue) and two-photon (red) event images. Cross-section rows 80–110 of the images as indicated by the blue dotted lines on the image in Fig. 5. For each column in the selected rows the mean value was calculated for the one-event image and the two-event image. These mean values were normalised independently by dividing the maximum value for each respective cut as to allow representation on the same scale. All events registered as per our described analysis are present in the images and no background subtraction has been performed.

**Figure 5:**
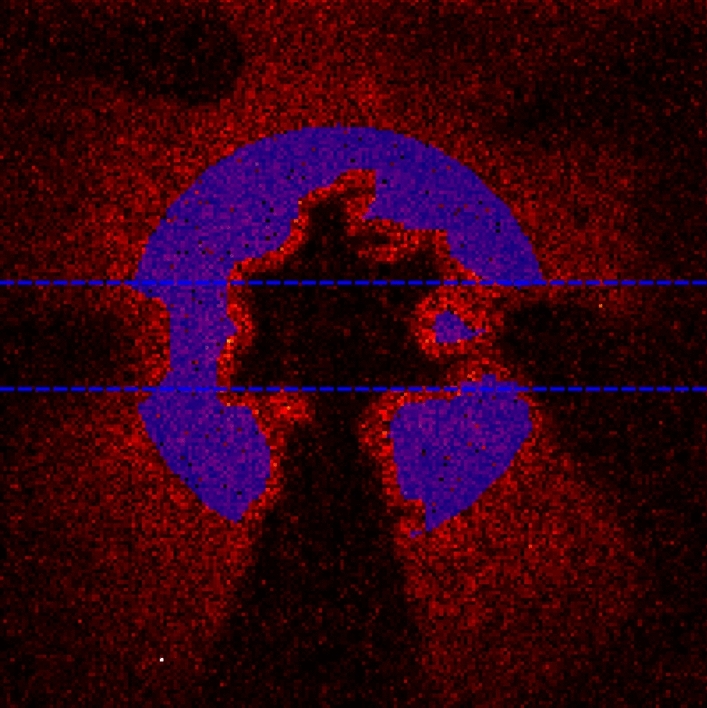
Region of interest. The region over which the metrics are calculated are contained within the blue circle which defines the extent of the SPDC beam. This region contains 5103 pixels over which the values for the fill factor and the 2/1 ratios are calculated. The blue dotted lines indicate the rows 80–110 which are averaged over in the cross-section graphs.

With the ability to distinguish the number of events in each pixel per frame images consisting of only one-photon events or two-photon events were obtained. One-photon and two-photon event images of a binary star target obtained over 10,000 frames may be seen in Fig. 4, along with an image intensity cross-section plot generated by averaging over the rows indicated in Fig. 5. The one-photon event image has a larger number of events and displays a smoother intensity distribution across the SPDC beam. However, a greater number of events occur in the masked portions of the image consisting of single photon events which reduces the contrast between the bright and dark portions of the image when compared to the image consisting of two-photon events. These events in the dark portions of the image are a result of camera noise events, of which there are fewer two-photon events than there are one-photon camera noise events. The reduction in contrast in the one-photon event image compared to the two-photon image due to these camera noise events can be seen in the intensity cross-sections in Fig. 4c.

### Photon-pair imaging

**Figure 6:**
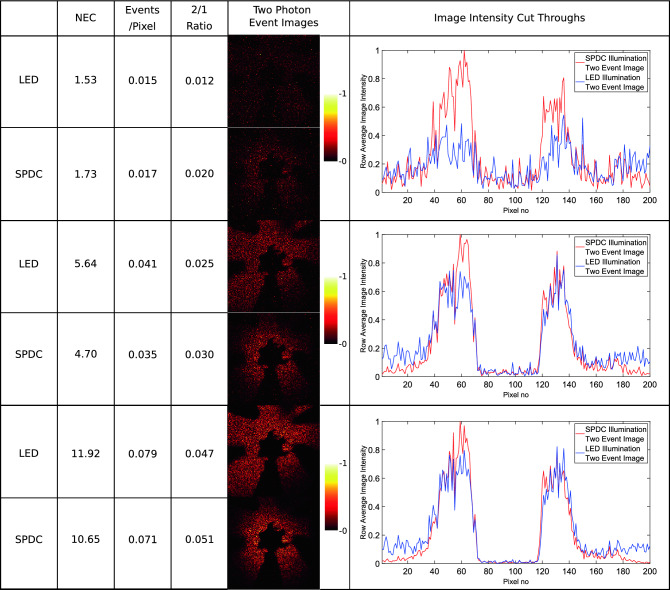
Comparative images between LED and SPDC illumination at increasing light levels. Two-photon event images acquired using LED and SPDC illumination over 10,000 frames. The ratio of two-photon events to one-photon events (2/1 event ratio) calculated from the rejected one-photon events is shown for each image. Increased two-photon to one-photon event ratios are seen when using the SPDC pair source. Also shown are the events per pixel per frame for the respective images. For the purposes of visualisation each pair of images are normalised to the same scale. This is performed using the maximum count within each image pair. Also shown are intensity cross-section plots for the displayed image pairs from the rows shown in Fig. 5. All events registered as per our described analysis are present in the images and no background subtraction has been performed.

**Figure 7:**
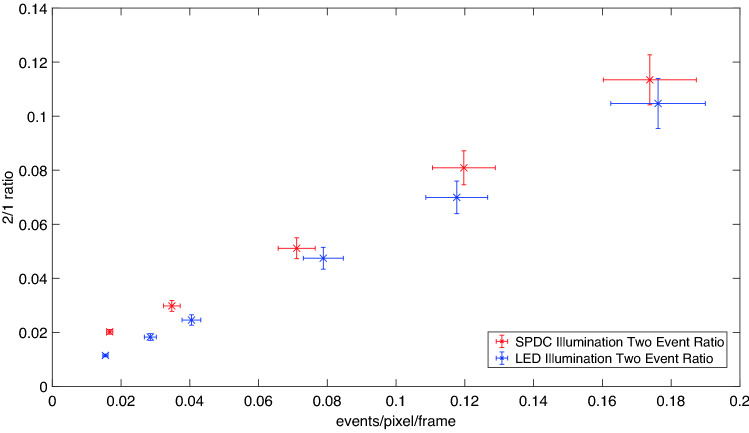
Plot of the 2/1 photon event ratio against illumination level Plot of the two to one-photon event ratio against the events per pixel per frame across a range of light levels. The error bars represent the standard error on the mean for the 2/1 ratio and the events per pixel per frame calculated over 10 blocks of 1000 frames.

To compare the performance of the imaging system at selecting correlated photon-pairs two-photon event images for the downconversion source are compared with a LED source. These comparison images for increasing illumination levels can be seen in Fig. 6. Our illumination levels are set by a reference light level, where the number of illumination events is equal to the number of camera noise events, termed noise equivalent counts (NEC). Also shown is the ratio of two-photon events to one-photon events (2/1 ratio) calculated from the number of two-photon events and corresponding number of ‘rejected’ one-photon events for each image. This is a key metric in the performance of the system as it represents the ability of the imaging system to select downconverted pair events. Illumination with SPDC shows an increased ratio of two-photon to one-photon events compared to the LED source. Figure 7 shows the two-photon to one-photon event ratio across a greater range of illumination levels. It can be seen that at lower illumination levels the advantage in the two-photon to one-photon event ratio is greater, whereas at higher illumination levels the advantage is reduced. For the SPDC illumination increasing the illumination levels will not only increase the number of two-photon events that correspond to photon-pairs, but also will result in an increased number of ‘accidental’ two-photon events. These accidental two-photon events arise from combinations of camera noise—camera noise event pairs and single photon—camera noise event pairs as opposed to the ‘true’ photon-pair events originating from photon-pairs generated by SPDC. The proportion of total two-photon events that correspond to ‘true’ photon-pair events is maximised at illumination levels where the number of photon events is approximately equal to the number of detector noise events^29^.

The improved two-photon to one-photon event ratio provides an increased contrast in images where the object was illuminated by the SPDC source compared to the LED. This can be seen from the corresponding cross section image intensity plots in Fig. 6. The enhancement in contrast follows the improvement in two-photon to one-photon event ratios, with greater contrast improvements seen at lower light levels. A maximum improvement in the 2/1 photon event ratio by a factor of 1.66 is observed for the pair of images obtained under an illumination level of $\sim$ 1.5 to 1.7 NEC.

The key performance parameter in our experiment is the ratio between the number of pixels containing a one-photon event, whether it be signal or noise, and the number of pixels containing two-photon events for the LED and SPDC photon-pair source respectively. Considering first the limit of zero detector noise, and assuming a low illumination level with the number of events per pixel per frame $E_{Tot,Photon} \ll 1$, then for a classical LED source emitting photons with random spatial positions the fraction of pixels containing a one-photon event is simply $E_{1,Photon} \approx E_{Tot,Photon}$ and the fraction containing a two-photon event is $E_{2,Photon} \approx \frac{1}{2} E_{1,Photon}^2$. This would give a two-photon event to one-photon event ratio of $\approx \frac{1}{2} E_{Tot,Photon}$.

Replacing the LED with the SPDC photon-pair source at the same illumination intensity, and assuming that the position correlation between the pair of photons is small compared to the pixel size, then the increase in the two event to one event ratio depends upon the efficiency of detecting both photons in a pair, the heralding efficiency, $\eta$. The heralding efficiency therefore would determine the upper bound of the performance of the photon-pair source when compared to the LED. Under the conditions of a low heralding efficiency the two-photon event to one-photon event ratio then becomes $\approx \frac{\eta }{2} + \frac{E_{Tot,Photon}}{2}(1-\eta )^{2}$.

The camera noise of the detector is gaussian in nature and does not hold the above relation in that the number of events corresponding to two-photon events $E_{2,Noise}$ is not equivalent to half the number of one-photon events squared $\approx \frac{1}{2} E_{1,Noise}^2$. Therefore in the presence of detector noise the two sources of detector events must be separated. The expected additional number of events corresponding to accidental instances of two-photon events in the case of the uncorrelated illumination is modified to $\frac{1}{2}E_{1,Photon}^{2}+ E_{1,Photon}E_{1,Noise}+E_{2,Noise}$.

It follows that the largest difference in the two-photon and one-photon event ratios between the LED source and SPDC photon-pair source will be obtained when operating at a low number of total events per pixel per frame, $E_{Tot,Photon}$, and a high heralding efficiency, $\eta$. In practice, the lower limit on $E_{Tot,Photon}$ is set so as to be higher than the residual readout noise of the sensor array in order to prevent images becoming dominated by camera noise. The practical heralding efficiency is determined by multiple experimental factors such as the quantum efficiency of the sensor and losses due to various components in the imaging system. In our case the greatest difference in the two-photon to one-photon event ratio between the LED and SPDC source is obtained at low illumination levels of $\sim$ 1.5 to 1.7 NEC where the number of accidental two-photon events is minimised. At this illumination level the number of optical events is approximately $1.5 \times$ that of the camera noise events meaning the total events per pixel per frame is set at $E_{Tot,Photon} \approx 0.016$ for a dark event rate of 0.006 events per pixel per frame. Using the SPDC photon-pair source results in an increase in the two-photon event to one-photon event ratio by a factor of 1.66 when compared to the LED photon source. In the future the two-photon to one-photon ratio obtained using a SPDC source could be improved through an increase in the heralding efficiency of the optical system, $\eta$, or a decrease in the readout noises of future CMOS sensors.

## Discussion

We have reported a new method for the detection of spatially correlated photon-pairs from a SPDC source with a photon number resolving camera. With this imaging system images an improved ratio of two-photon events to one-photon events are obtained with a quantum SPDC photon-pair source when compared with a LED source across a range of illumination levels. Resulting images consisting only of two-photon events show a maximum improvement in the two-photon event to one-photon event ratio for the SPDC source compared to the LED source when operating at a low illumination level so as to minimise the contribution of accidental event-pairs. The presence of an enhancement in the 2/1 ratio for the quantum photon-pair source demonstrates the validity of the concept and is encouraging for the application of photon counting cameras in future quantum imaging applications.

Quantum enhanced imaging experiments that require the detection of both photons in a photon-pair to realise an enhancement could benefit from the ability to distinguish one-photon events from two-photon events using the technology and methods described here. In an experimental configuration that places the detector in the image plane of the downconversion crystal the most tightly correlated photon-pairs arrive in the same pixel and this information is not easily recovered in the case of using a conventional CCD or CMOS detector technology for which it is difficult to distinguish between one-photon events and two-photon events. The ability to distinguish the number of photon events allows an increased amount of information to be recovered on a per frame basis thereby allowing increased efficiency and reduced acquisition times.

Further improvements in the performance are set by the efficiency of photon-pair detection and camera readout noise, we expect improvements to these factors to yield more dramatic enhancements in the contrast of images. Higher detection efficiency could be achieved for example by creating down-converted photon-pairs at a shorter wavelength where the quantum efficiency of the sensor is higher. Furthermore, advancements in CMOS camera technology should result in lower sensor readout noises. With more dramatic contrast enhancements it is expected that images could be acquired on fewer frames where it is only possible to reveal the object with a quantum pair source. Such an experiment approaching real time imaging could point to applications in low light level microscopy or covert target detection systems.

## Methods 

The pump beam source used here was a JDSU xCyte CY-355-150 Nd:YAG laser with quasicontinuous output at $355 \,\text {nm}$ output at $150 \,\text {mW}$, with a pulse repetition of $100 \pm 10 \,\text {Mhz}$ and pulse width of $>10 \,\text {ps}$. The spatial filter used to collimate and expand the pump consists a $50 \,\text {mm}$ lens, a $25 \mu \text {m}$ pinhole, and a $200 \,\text {mm}$ lens. The down-conversion source was a BBO non-linear crystal of dimensions $10 \,\text {mm } \times 10 \,\text {mm} \times 1 \,\text {mm}$ cut for type-I collinear phase-matched downconversion at degenerate wavelength of $710 \,\text {nm}$. The classical light source used is a Thorlabs LED M660D2 which emits light at $660 \,\text {nm}$. The camera used was a prototype Hamamatsu ORCA-Quest qCMOS camera cooled to − 20 °C, operated on ultra-quiet mode (rms readout noise $0.27e^{-}$) acquiring at $\sim 30 \,\text {fps}$ on an exposure time of 0.03 s which defines the temporal resolution of the system. Chroma T4551pxt dichroic mirrors with a cutoff wavelength of $455 \,\text {nm}$ and $98 \%$ transmission at $710 \,\text {nm}$ were used to remove the pump beam. The filter placed on the camera was a Chroma ET710/10m interference filter, with a $10 \,\text {nm}$ bandpass with a top-hat profile centred at $710 \,\text {nm}$ ($99 \%$ efficiency).
